# Medical Association of Georgia

**Published:** 1884-04-20

**Authors:** 


### *MEDICAL ASSOCIATION OF GEORGIA.*

The Medical Association of Georgia held its 35th Annual Meeting in Macon on 16th, 17th and 18th of April, 1884.

The address of welcome was eloquently delivered by W. F. Holt, M.D., of Macon, and responded to by Dr. L. B. Alexander, of Forsyth, on the part of the Association, in appropriate and beautiful language.

This was followed by the address of the retiring President, Dr. K. P.

Moore, of Macon, which was in good taste and highly complimentary to his successor, Dr. A. B. Calhoun, of Atlanta, who, on assuming the Chair, delivered an able and interesting address on the subject of "*School Hygiene, in Relation to its Influence on the Vision of Children.*"

Dr. J. P. Logan, Chairman of the Committee on Inebriate Asylum, reported that he had been uuable to obtain any encouragement from the Legislature on the subject.

Dr. H. V. M. Miller made some remarks, as Chairman of the Committee on Expert Testimony, unfavorable as to the hope of obtaining any legislation in respect to it, as also of the matter of anatomical procurement. The committees were, however, continued, and the duties recommitted.

Dr. Eugene Foster, of Augusta, presented touching tributes in honor of the following deceased members, to wit: Dr. E. W. Hunter, of Louisville, Sterling Eve, of Augusta, J. M. Carlton, of Athens, and L. D. Ford, of Augusta.

The Committee on Prize Essays reported no papers presented worthy of the prize, and the fund in hand for this purpose is held over and the Committee instructed to continue the offer, narrowing the essays to two or three subjects.

SECOND DAY.

The regular committees from the several districts reported only in part. The following only reported:

Dr. V. H. Talliaferro, Gynecology.

Dr. N. P. Jelks, on Praetice.

Dr. A. C. Perry, on Practice.

Dr. W. B. Roberts, on Surgery.

Dr. J. G. Gerardent, on surgery.

Dr. R J. Nunn, on Gynecology.

Volunteer papers were presented by the following gentlemen: Drs. T. S. Powell, Jno. Thad. Johnson and A. G. Hobbs, Atlanta; H. J. Williams, Macon. J. M. Hall, Augusta; Eugene Foster, Augusta; J. P. Stevens, Macon; W. B. Parks, Atlanta; J. W. Flanders, Wrightsville; H. McHatton, Macon; H. F. Scott, Atlanta; N. G. Gervenner, Macon; F. Ekland, of Stockholm; E. G. Ferguson, Macon; C. W. Hickman, Augusta; J. M. Gastonn, Atlanta; T. M. McIntosh, Thomasville; H. V. M. Miller; W. O. Daniel, Ballards.

Considerable discussion was had in reference to reducing the initiation fee from $3 oo to $2.oo, upon which a special committee was appointed to report at next meeting, consisting of Drs. Bizzell, Nunn and Talliaferro.

The banquet was held, on the night of the 17th, at the Brown House, in which Macon hospitality was well illustrated. It was a sumptuous and brilliant affair, more than a hundred doctors being present.

Much hilarity and good feeling prevailed. The following toasts were drunk:

The first toast was "The Medical Association of Georgia." Responded to by Dr. A. W. Calhoun. The Doctor spoke of the sometimes ingratitude of the world of people toward physicians, and concluded with a compliment to Macon and the reception accorded the Association.

The next toast was, "The National Board of Health."

"The Nation that guards her people's health,
Adds to herself both power and wealth."

This was appropriately responded to by Dr. H. V. M. Miller, of Atlanta.

The next toast was to "Hygeia."

> "Onward she moves! Disease and Death retire,
> And murmuring demons hate her and admire."

This should have been responded to by Dr. Taliaferro, but that gentleman was not present.

The next toast announced was "Our Medical College."

> "A little learning is a dangerous thing.
> Drink deep or taste not the Pierian spring."

Responded to in a happy manner by Dr. W. P. Nicolson, of Atlanta.

The toast to "Our Literary Colleges,"

> "Learning by studying must be won—
> 'Twas ne'er entailed from sire to son,"

was responded to by Dr. A. J. Battle, of Macon.

The toast to "Woman,"

> "Best gift to man from Heaven above,
> Whose mission here on earth is love,"

was responded to by Dr. McIntosh.

The officers elected for the ensuing year are as follows:

President—Dr. Eugene Foster, of Atlanta.

First Vice President—Dr. I. B. Roberts, of Sandersville.

Second Vice President—Dr. W. D. Bizzell, Atlanta.

Same Committee on Publication continued.

As we go to press, we have not yet obtained the names of the various committees which were appointed. We have the following from the Fifth District:

Section on Practice—W. D. Bizzell, J. S. Todd, W. A. J. Anderson.

Section on Surgery—W. P. Nicolson, R. H. Taylor, H. F. Scott.

Section on Gynecology—J. G. Earnest, T. K. Mitchell, G. H. Noble.

The next meeting will be at Savannah on the 3d Wednesday of April, 1885. The Orator appointed for the occasion was Dr. J. B. Baird, of Atlanta. The local Committee of Arrangements at Savannah are Drs. Thomas, Reid, Charlton, Duncan and Le Hardy.

The editors of this journal were both unavoidably absent from the Association, but from friends who were present we are assured that it was in all respects the most pleasant and most profitable meeting which has been held for many years.

